# The V Protein of Tioman Virus Is Incapable of Blocking Type I Interferon Signaling in Human Cells

**DOI:** 10.1371/journal.pone.0053881

**Published:** 2013-01-14

**Authors:** Grégory Caignard, Marianne Lucas-Hourani, Kevin P. Dhondt, Jean-Louis Labernardière, Thierry Petit, Yves Jacob, Branka Horvat, Frédéric Tangy, Pierre-Olivier Vidalain

**Affiliations:** 1 Unité de Génomique Virale et Vaccination, Centre National de la Recherche Scientifique (CNRS) URA-3015, Virology Department, Institut Pasteur, Paris, France; 2 Institut National de la Santé et de la Recherche Médicale (INSERM) U758, Ecole Normale Supérieure de Lyon, Lyon, France; 3 University of Lyon 1, Lyon, France; 4 Zoo de La Palmyre, Les Mathes, France; 5 Unité de Génétique, Papillomavirus et Cancer Humain, Virology Department, Institut Pasteur, Paris, France; University of Missouri-Columbia, United States of America

## Abstract

The capacity of a virus to cross species barriers is determined by the development of *bona fide* interactions with cellular components of new hosts, and in particular its ability to block IFN-α/β antiviral signaling. Tioman virus (TioV), a close relative of mumps virus (MuV), has been isolated in giant fruit bats in Southeast Asia. Nipah and Hendra viruses, which are present in the same bat colonies, are highly pathogenic in human. Despite serological evidences of close contacts between TioV and human populations, whether TioV is associated to some human pathology remains undetermined. Here we show that in contrast to the V protein of MuV, the V protein of TioV (TioV-V) hardly interacts with human STAT2, does not degrade STAT1, and cannot block IFN-α/β signaling in human cells. In contrast, TioV-V properly binds to human STAT3 and MDA5, and thus interferes with IL-6 signaling and IFN-β promoter induction in human cells. Because STAT2 binding was previously identified as a host restriction factor for some *Paramyxoviridae*, we established STAT2 sequence from giant fruit bats, and binding to TioV-V was tested. Surprisingly, TioV-V interaction with STAT2 from giant fruit bats is also extremely weak and barely detectable. Altogether, our observations question the capacity of TioV to appropriately control IFN-α/β signaling in both human and giant fruit bats that are considered as its natural host.

## Introduction

**Figure 1:**
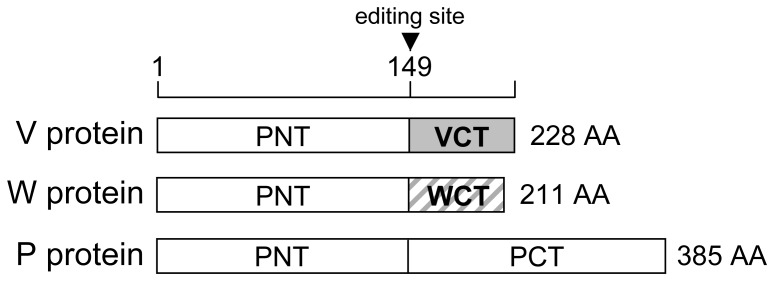
The gene P of TioV encodes for three proteins: V, W and P. Whereas conventional transcription and translation lead to the expression of TioV-V, co-transcriptional insertion of one G residue at the editing site by the viral RNA polymerase leads to the expression of a chimeric protein called W. Insertion of two G residues can also occur during transcription, thus leading to the expression of the phosphoprotein P.

*Paramyxoviridae* is a family of viruses with a negative-sense RNA genome that includes important human pathogens like measles virus (MeV), human parainfluenza virus type 3 (hPIV3), and human respiratory syncytial virus (hRSV) [1]. As demonstrated by phylogenic studies, these human pathogens emerged from zoonotic events that occurred hundreds or thousands of years ago [2]. Novel *Paramyxoviridae* have also emerged recently because of major ecological changes [3]. Deforestation in tropical areas has destroyed the natural habitat of fruit bat species, forcing them to live in the vicinity of human settlements. These close contacts are responsible, in Southeast Asia and Australia, for the emergence of highly pathogenic *Paramyxoviridae* in local human populations such as Nipah virus [4]. While searching for traces of this virus in urine samples from giant fruit bats of the *Pteropus* genus, Kaw Bing Chua and collaborators have isolated another previously unknown *Paramyxoviridae* from *Rubulavirus* genus that was named Tioman virus (TioV) [5]. Its negative-sense single-strand RNA genome encodes for six structural proteins that directly participate in viral replication and/or particle assembly. In addition, the P locus encodes for two non-structural proteins, V and W (Figure 1), which are considered as essential virulence factors by homology with other rubulaviruses like mumps virus (MuV). Some neutralizing antibodies against TioV have been found in serum samples from local inhabitants, suggesting close contacts with this virus [6]. Nevertheless, and despite its ability to infect human cells *in vitro* [5], [7], whether TioV is associated to some human pathology remains undetermined. Furthermore, biochemical and functional properties of TioV proteins, in particular the two non-structural factors V and W, remain poorly characterized.

*Paramyxoviridae* V proteins are potent and multifunctional inhibitors of type I interferon (IFN-α/β) pathway, which is the core component of antiviral immune response in mammals [8], [9]. First, *Paramyxoviridae* V proteins interact with two cellular proteins involved in cytoplasmic sensing of viral RNA molecules, MDA5 and LGP2, and thus impair IFN-α/β expression in infected cells [10], [11], [12], [13]. In addition, *Paramyxoviridae* V proteins interfere with cell signaling downstream of IFN-α/β receptor, but each genus within the family exhibits specific mechanisms of inhibition [14]. For rubulaviruses, the molecular mechanism underlying the inhibition of IFN-α/β signaling has been well documented for parainfluenza virus type 5 (PIV5), mumps virus (MuV) and human parainfluenza virus type 2 (hPIV2). Once secreted, IFN-α/β bind to membrane receptor IFNAR1/IFNAR2c, and trigger the activation of STAT1 and STAT2 transcription factors that together induce the expression of a large antiviral gene cluster. Rubulavirus V proteins inhibit this pathway by interacting and inducing STAT protein polyubiquitinylation and degradation through the recruitment of an E3 ubiquitin ligase complex composed of DDB1, Cul4A and Rbx/Roc1 subunits [15], [16], [17], [18], [19], [20], [21], [22], [23], [24]. Interestingly, MuV and PIV5 V proteins require cellular STAT2 as an adaptor to recruit and eliminate STAT1. In contrast, hPIV2 V protein uses STAT1 as an adaptor to target STAT2 for degradation (although in some cases it can directly target STAT1 for degradation) [23], [25], [26], [27]. MuV-V has also evolved a distinct binding interface to recruit directly STAT3 for ubiquitination and degradation [28], [29], resulting in the inhibition of IL-6 signaling, another pathway involved in the host antiviral response. Alike MuV-V, the V protein of TioV (TioV-V) has been shown to bind MDA5 and LGP2, thus inhibiting IFN induction by viral RNA molecules [10], [13]. However, whether TioV-V is also able to inhibit IFN-α/β signaling pathway by targeting STAT1/2 proteins for proteasomal degradation has not been addressed. Surprisingly, preliminary data obtained from a high-throughput functional screen that we previously performed suggested that the V protein of TioV is defective for the inhibition of IFN-α/β signaling [30]. This led us to study in further details TioV-V capacity to block this signaling pathway in human cells.

## Results

### TioV Infection Strongly Induces IFN Signaling in Human Cells

**Figure 2:**
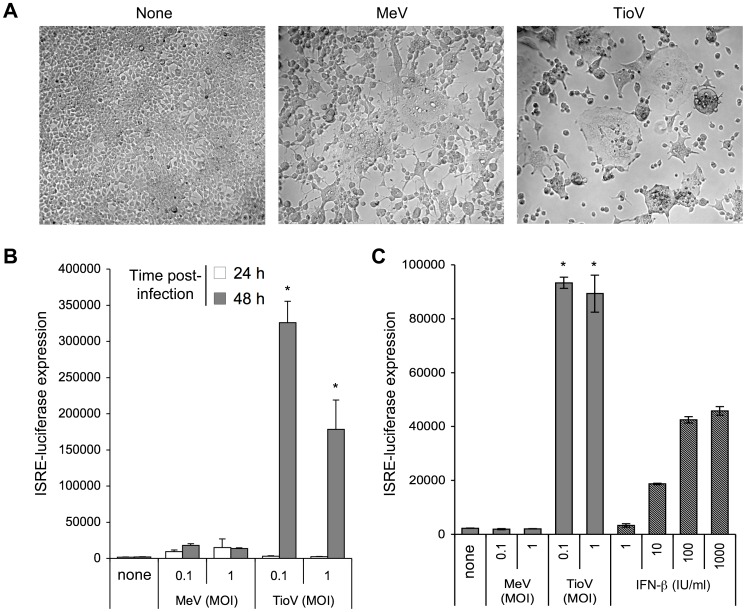
Activation of ISRE-dependent gene expression by MeV or TioV infection. HEK-293 cells stably transfected with an ISRE-luciferase reporter gene (STING-37 reporter cell line) were infected with MeV or TioV. (**A**) Bright field microscopy of cell cultures at 48 h post-infection (MOI  = 1). (**B**) Luciferase expression was determined at 24 h and 48 h post-infection. (**C**) Culture supernatants from (B) were collected at 48 h post-infection, clarified by centrifugation, UV-inactivated and added to culture wells containing STING-37 cells. Alternatively, culture medium was supplemented with increasing doses of recombinant IFN-β. After 24 h, luciferase expression was determined. Luciferase activity in culture supernatants from (B) was below 1,400 luciferase activity units (data not shown). Experiment was performed in triplicates, and data represent means ± SD. *indicates that differences observed between MeV and TioV-infected cells were statistically significant (p-value <0.01).

Previous studies have established the infection of human cells by TioV [5], [7], but whether this induced IFN signaling required further investigations. We have recently established a HEK-293 cell line with a luciferase reporter gene under control of five IFN-stimulated response elements (ISRE). This reporter cell line was used to determine the activation of IFN signaling pathway in human cells upon TioV infection. In parallel, cells were infected with measles virus (MeV). Indeed, this virus encodes well-known virulence factors, including P, V and C proteins, which aim at controlling IFN signaling induction and antiviral response. The observation of cytopathic effects and formation of numerous syncytia at 48 h of culture confirmed cellular infection by TioV and MeV (Figure 2A). Although MeV-infected cells expressed moderate levels of luciferase, TioV infection was characterized by a strong induction of the ISRE-luciferase reporter gene at 48 h post-infection (Figure 2B), which reflects a potent activation of IFN signaling. This suggests that TioV is incapable of blocking IFN signaling, but also fails to control IFN production. Indeed, we found that UV-inactivated supernatant from TioV-infected HEK-293 cells strongly induced ISRE-luciferase expression when applied to fresh reporter cells, whereas supernatant from MeV-infected cells did not (Figure 2C). This is surprising since TioV-V protein expression was previously shown to block MDA5 and RIG-I signaling through interactions with MDA5 and LGP2, respectively [10], [13]. However, many cellular components involved in viral sensing and IFN production are IFN-inducible. As a consequence, some defect in TioV-V capacity to block IFN signaling could translate into a robust expression of IFN at later time-points of the infection, thus explaining our results [31]. Altogether, these observations motivated the functional analysis of TioV-V protein and its interactions with the IFN signaling pathway.

### The V protein of TioV is Incapable of Blocking IFN Signaling in Human Cells

**Figure 3:**
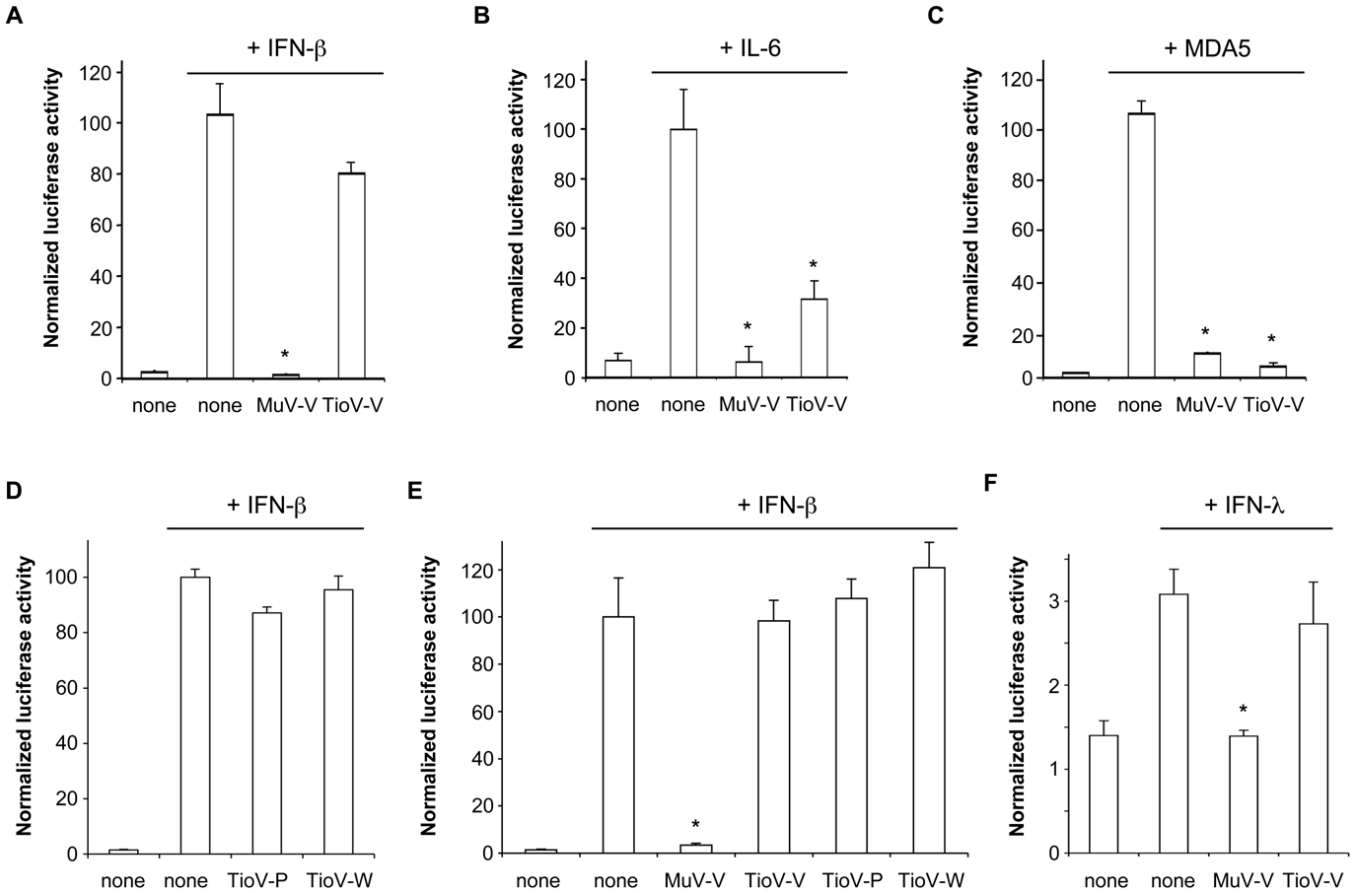
TioV-V inhibits IFN-β promoter activation by MDA5 and IL-6 signaling, but not IFN-α/β signaling. (**A**) HEK-293T cells were co-transfected with reporter plasmid pISRE-Luc (300 ng/well), pRL-CMV reference plasmid (30 ng/well), and pCI-neo-3xFLAG expression vectors encoding for 3xFLAG alone or fused to MuV-V or TioV-V (300 ng/well). After 24 h, recombinant IFN-β was added at 200 IU/ml. After an additional 24 h, relative luciferase activity was determined. (**B**) HEK-293T cells were co-transfected with reporter plasmid pSTAT3-Luc (300 ng/well), pRL-CMV (30 ng/well), and expression vectors encoding 3xFLAG-tagged MuV-V or TioV-V (300 ng/well). At 24 h post-transfection, recombinant IL-6 was added at 10 ng/ml. After an additional 24 h, relative luciferase activity was determined. (**C**) HEK-293T cells were co-transfected with IFN-β-pGL3 reporter plasmid (300 ng/well), pRL-CMV (30 ng/well), expression vectors encoding 3xFLAG-tagged MDA5 (300 ng/well) and MuV-V or TioV-V (300 ng/well). After 48 h, relative luciferase activity was determined. (**D**) Experiment was performed as in (A), but cells were co-transfected with expression vectors encoding 3xFLAG-tagged TioV-P or TioV-W. (**E**) Experiment was performed as in (A), but cells were co-transfected with pCI-neo expression vector, either empty or encoding untagged MuV-V, TioV-V, TioV-P or TioV-W. (**F**) Experiment was performed as in (A), but cells were stimulated with recombinant IFN-λ1 at 50 ng/ml. All experiments were performed in triplicates, and data represent means ± SD. *indicates that differences observed relative to controls (none) with IFN-β, IL-6, MDA5 or IFN-λ were statistically significant (p-value<0.01).

TioV-V was first tested for its ability to block IFN-β signaling downstream of its receptor, and compared to MuV-V which is known to block this pathway through STAT2 binding and STAT1 targeting for proteasomal degradation. Plasmids encoding for MuV-V or TioV-V were co-transfected in HEK-293T human cells together with an ISRE-luciferase reporter plasmid, and stimulated after 24 hours with 200 IU/ml of recombinant IFN-β. TioV-V was unable to impair ISRE activation by IFN-β, whereas MuV-V efficiently inhibited signal transduction as expected (Figure 3A). To demonstrate that TioV-V is specifically defective for this function, we compared MuV-V and TioV-V for their ability to block IL-6 signaling by using a STAT3-dependent luciferase reporter gene. Although MuV-V was more efficient than TioV-V, both viral proteins impaired luciferase expression in IL-6 stimulated cells (Figure 3B). We also compared MuV-V and TioV-V for their capacity to block IFN-β promoter induction by MDA5 overexpression. As shown in Figure 3C, MuV-V and TioV-V equally inhibited this pathway in agreement with literature [10].

Altogether, these results demonstrate that both MuV-V and TioV-V inhibit MDA5-dependent IFN-β induction and IL-6 signaling in human cells whereas cell signaling downstream of IFN-α/β is only impaired by MuV-V. In addition to V, the P gene of TioV also encodes for the phosphoprotein P, which is part of the viral replication complex, and W of which function is undefined. Although a previous report showed that STAT1 can be only degraded by the full-length PIV5 V protein, excluding the involvement of the phosphoprotein P [16], we tested if TioV-P or TioV-W could substitute for TioV-V and block IFN-α/β signaling. However, neither TioV-P nor TioV-W inhibited ISRE activation when stimulating cells with recombinant IFN-β (Figure 3D). As a control, inhibition of IFN-α/β signaling was also determined when expressing viral proteins without a N-terminal 3xFLAG tag to verify that it did not interfere with their function (Figure 3E). Results obtained confirmed our previous conclusions, showing that known products of the P gene, and in particular TioV-V, were unable to block IFN-α/β signaling. Finally, and because TioV has been previously shown to induce type III interferons (IFN-λs) in primary bat splenocytes [32], we determined if TioV-V was capable of blocking this signaling pathway. Although type III interferons bind a specific membrane receptor called IFNLR1/IL10R2, which is distinct from IFN-α/β receptor (IFNAR1/IFNAR2c), these cytokines activate STAT1/2 phosphorylation and induce expression of ISRE-regulated genes alike IFN-α/β. In our reporter system, recombinant IFN-λ only induced a weak expression of the ISRE-luciferase gene, but this induction was blocked by MuV-V whereas TioV-V was unable to do so (Figure 3F). This demonstrated that unlike MuV-V, TioV-V is incapable of targeting components of both IFN-α/β and IFN-λ signaling pathways.

**Figure 4:**
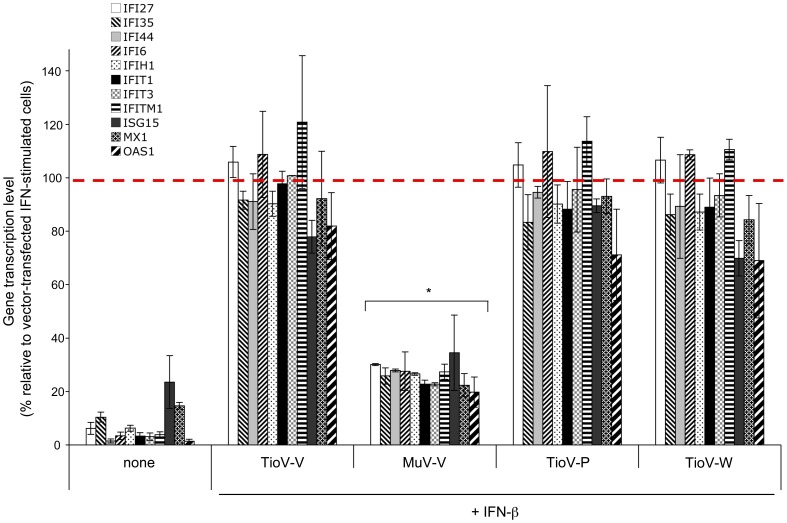
Expression of IFN-inducible genes is impaired by MuV-V but not TioV-V expression. HEK-293T cells were transfected with pCI-neo-3xFLAG expression vectors encoding for 3xFLAG alone or fused to TioV-V, MuV-V, TioV-P or TioV-W (500 ng/well). 24 h after transfection, cells were left unstimulated or stimulated with 200 IU/ml of recombinant IFN-β. After 24 h of culture, total RNAs were extracted, and expression levels of indicated genes were quantified by qRT-PCR. For each gene, data were normalized so that 100% corresponds to cells transfected with pCI-neo-3xFLAG empty vector and stimulated with recombinant IFN-β (dotted red line). Experiment was performed twice and data represent means ± SD. *indicates that differences observed with MuV-V relative to controls cells transfected with 3xFLAG alone and stimulated with IFN-β were statistically significant (p-value<0.05).

To further demonstrate that TioV-V is unable to block IFN-α/β signaling in human cells, we quantified the induction of several IFN-inducible genes in cells expressing either MuV-V or TioV-V. HEK293-T cells were transfected with expression plasmids encoding for TioV-V, MuV-V, TioV-P or TioV-W, and then stimulated with IFN-β. Expression of eleven IFN-inducible genes, including IFI27, IFI35, IFI44, IFI6, IFIH1, IFIT1, IFIT3, IFITM1, ISG15, MX1, and OAS1, was determined by quantitative RT-PCR. As shown in Figure 4, gene expression was strongly inhibited by MuV-V, whereas TioV-V, TioV-P or TioV-W had no effect. Altogether, we demonstrated that in contrast to MuV-V, TioV-V or known products of the P gene like TioV-P and TioV-W are unable to block IFN-α/β signaling in human cells. This suggested that TioV-V is deficient for targeting specific cellular proteins, in particular host factors involved in IFN-α/β signaling events.

### TioV-V and MuV-V both Interact with Human MDA5, LGP2 and STAT3

**Figure 5:**
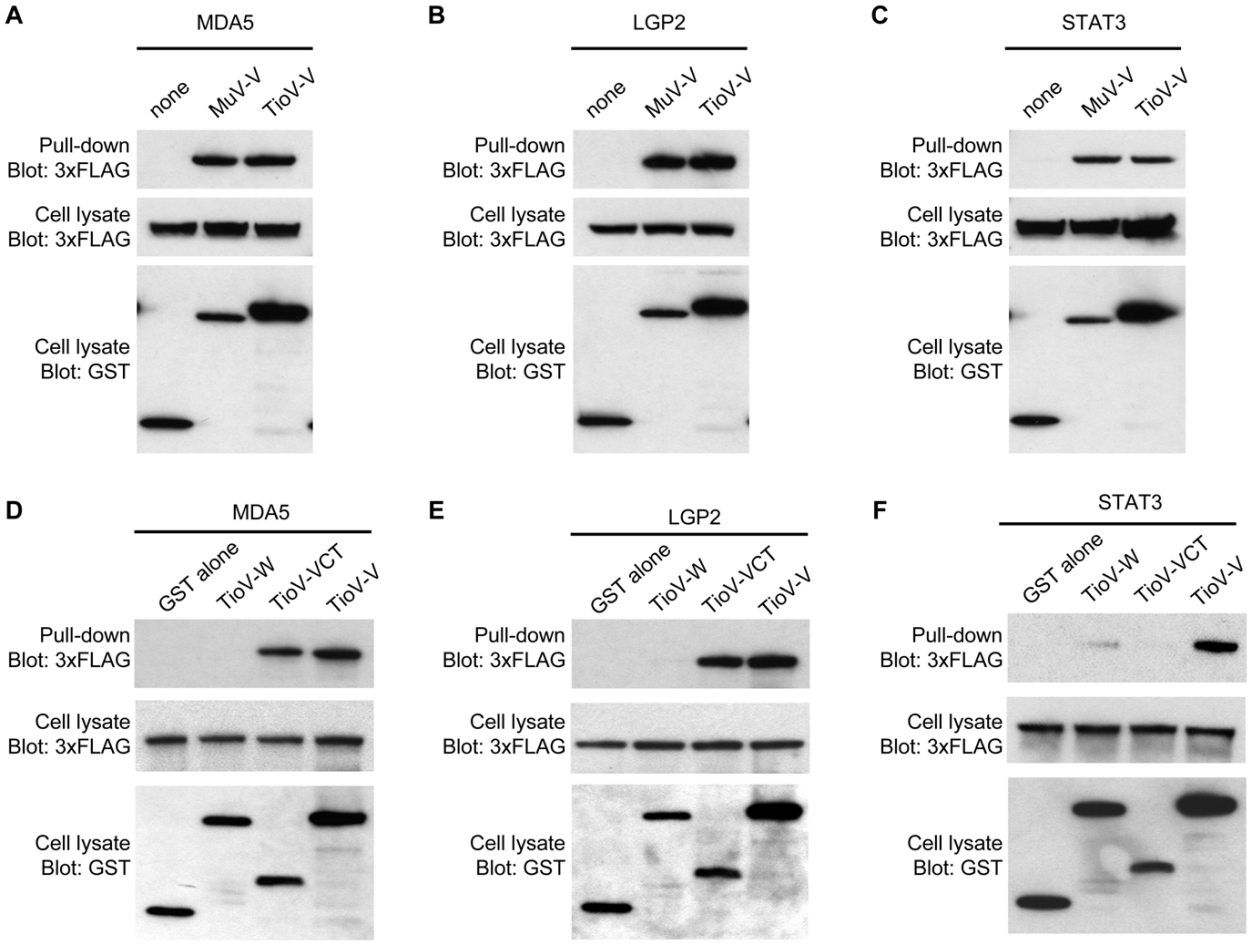
TioV-V binds MDA5, LGP2, and STAT3. (**A–C**) HEK-293T cells were co-transfected with expression vectors encoding GST alone or fused to MuV-V (**A–C**), TioV-V (**A–F**), TioV-W (**D–F**) or TioV-VCT (**D–F**) (500 ng/well), and pCI-neo-3xFLAG expression vectors (300 ng/well) encoding for 3xFLAG-tagged human MDA5 (**A** and **D**), LGP2 (**B** and **E**), STAT3 (**C** and **F**). Total cell lysates from transfected cells were prepared at 48 h post-transfection (cell lysate; middle and lower panels), and protein complexes were assayed by pull-down using glutathione-sepharose beads (GST pull-down; upper panel). 3xFLAG- and GST-tagged proteins were detected by immunoblotting.

The functional study described above was performed in parallel to a detailed analysis of TioV-V interaction profile with cellular proteins. First, TioV-V was used as bait in the yeast two-hybrid system to screen a human spleen cDNA library. The screen was performed at saturation with a 10-fold coverage of the library (50×10^6^ diploids), and positive yeast colonies growing on selective medium were analyzed by PCR and sequencing to identify binding partners of TioV-V (data not shown). Using this protocol, we identified MDA5, LGP2 and STAT3 as direct interactors of TioV-V. The interactions with MDA5 and LGP2 were previously reported [10], [13]. In contrast, STAT3 binding is new for TioV-V, although it was previously described for MuV [23], . To validate these interactions in human cells, GST-tagged TioV-V or MuV-V were co-expressed in HEK-293T cells with expression vectors encoding 3xFLAG-tagged MDA5, LGP2 or STAT3 and 48 h later, viral proteins were purified with glutathion-sepharose beads. MDA5, LGP2 and STAT3 co-purified with TioV-V protein, and results were equivalent to those observed with MuV-V (Figure 5A, B and C). To further establish the specificity of these interactions, we determined MDA5, LGP2 and STAT3 binding either to TioV-W, that shares its N-terminal region with TioV-V, or to the C-terminal VCT region that is specific of TioV-V. MDA5 and LGP2 only co-purified with TioV-V or the VCT region, thus demonstrating that the C-terminal region of TioV-V was sufficient to mediate these two interactions (Figure 5D and E). In contrast, STAT3 efficiently co-purified with TioV-V and some weak interaction was detected with TioV-W (Figure 5F). Thus, full-length TioV-V was required for a strong interaction with STAT3. Nevertheless, the weak interaction detected with TioV-W suggested that the N-terminal region is essential in agreement with a previous report showing that a single point mutation within the N-terminal region of MuV-V could prevent STAT3 binding [23], [28], [29]. Altogether, binding to MDA5 and STAT3 provide molecular basis to functional data presented in Figure 3B and 3C.

### As Opposed to MuV-V, TioV-V does not Efficiently Bind STAT2, Fails to Induce STAT1 Degradation, and does not Impair STAT1 Nuclear Translocation in IFN-β-stimulated Cells

**Figure 6:**
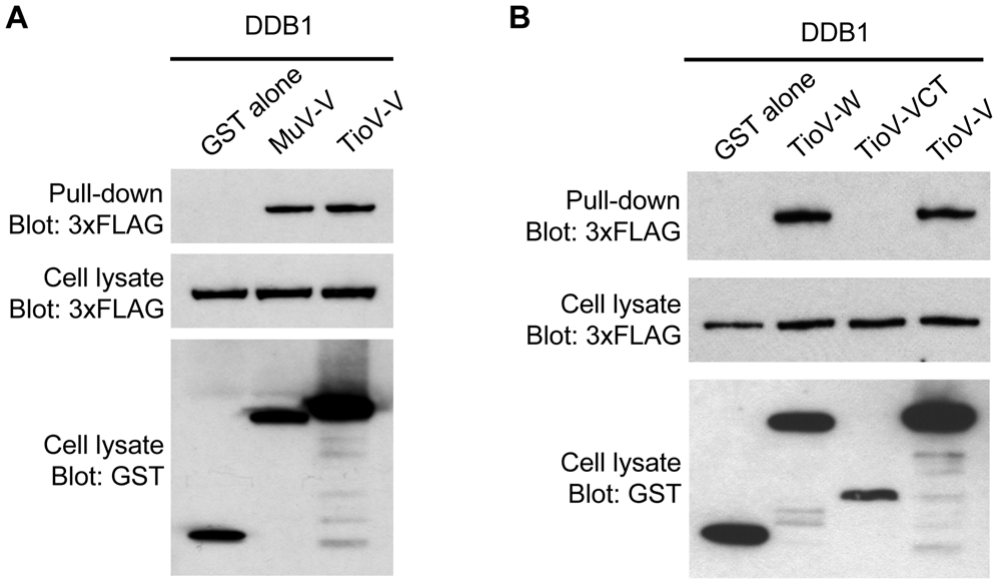
TioV-V binds DDB1. (A–B) HEK-293T cells were co-transfected with expression vectors encoding GST alone or fused to MuV-V (**A**), TioV-V (**A–B**), TioV-W (**B**) or TioV-VCT (**B**) (500 ng/well), and pCI-neo-3xFLAG expression vectors (300 ng/well) encoding for 3xFLAG-tagged human DDB1 (**A–B**). Total cell lysates from transfected cells were prepared at 48 h post-transfection (cell lysate; middle and lower panels), and protein complexes were assayed by pull-down using glutathione-sepharose beads (GST pull-down; upper panel). 3xFLAG- and GST-tagged proteins were detected by immunoblotting.

To block cell signaling downstream of IFN-α/β receptor, MuV-V relies on direct interactions with STAT2 and DDB1 to recruit and degrade STAT1 [15], [16], [17], [18], [20], [21], [22], [23], 24. Since TioV-V is unable to impair IFN-α/β signaling in human cells, we tested its ability to interact with human DDB1 and STAT2. We also tested TioV-V interaction with STAT1. Indeed, we cannot exclude the possibility that TioV-V binds directly STAT1 rather than STAT2 alike the V protein of hPIV2. First, GST-tagged TioV-V or MuV-V were co-expressed in HEK-293T cells with 3xFLAG-tagged DDB1. After 48 h, viral proteins were purified with glutathion-sepharose beads, and 3xFLAG-tagged DDB1 was revealed by western-blot analysis. As shown in Figure 6A, human DDB1 interacted with MuV-V and TioV-V, suggesting that both viral proteins can recruit the E3 ubiquitin ligase machinery in human cells. To further establish the specificity of this interaction, we determined DDB1 binding either to TioV-W or TioV-VCT as described above. In agreement with structural data showing that DDB1 binding peptide is localized within the N-terminal region of PIV5-V [33], [34], TioV-V and TioV-W but not TioV-VCT interacted with this cellular factor (Figure 6B).

**Figure 7:**
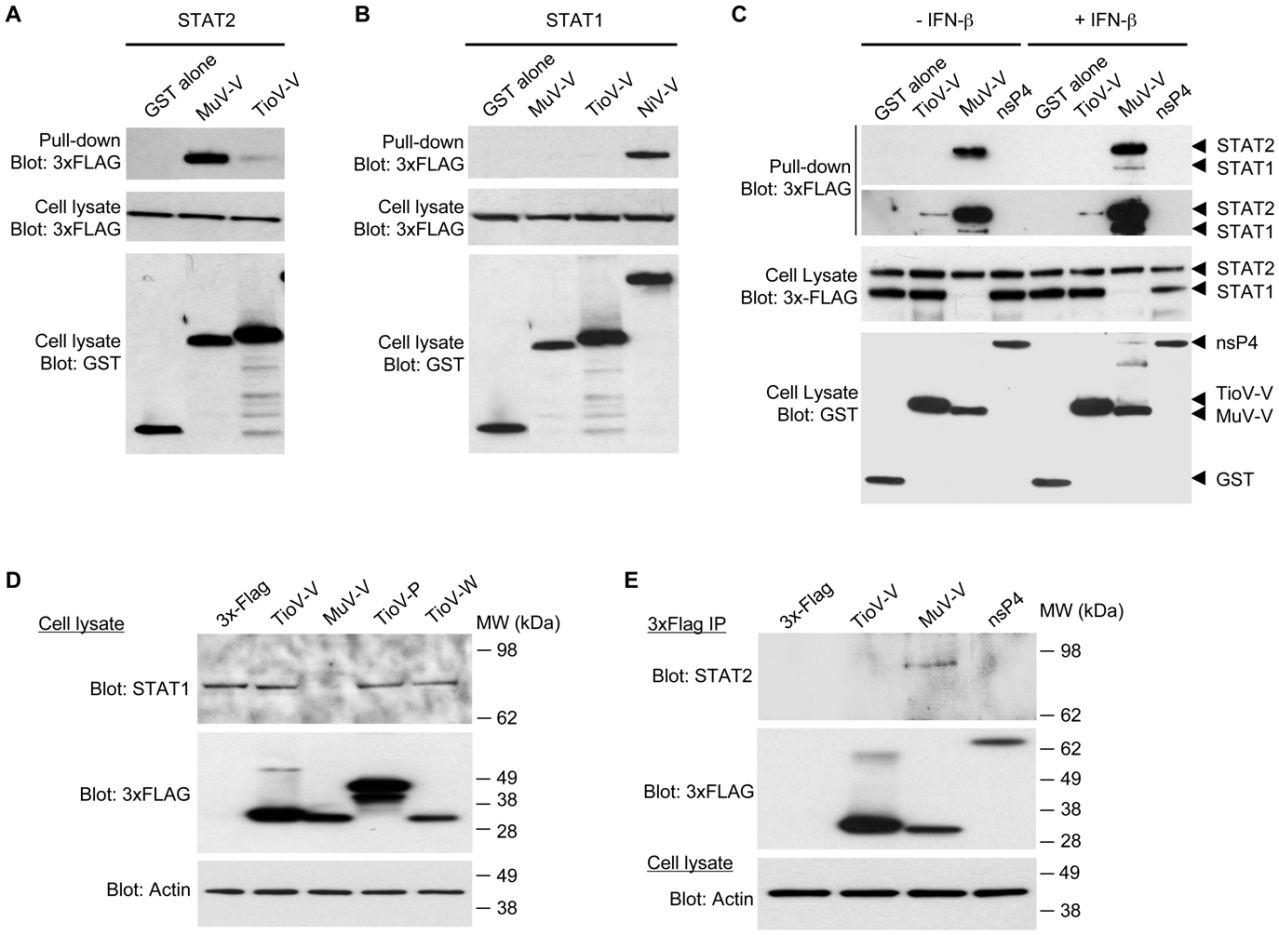
TioV-V fails to interact with human STAT2 and does not induce STAT1 degradation. (**A–B**) HEK-293T cells were co-transfected with expression vectors encoding GST alone or fused to MuV-V, TioV-V (**A–B**) or NiV-V (**B**) (500 ng/well), and pCI-neo-3xFLAG expression vectors (300 ng/well) encoding for 3xFLAG-tagged human STAT2 (**A**) or STAT1 (**B**). Total cell lysates from transfected cells were prepared at 48 h post-transfection (cell lysate; middle and lower panels), and protein complexes were assayed by pull-down using glutathione-sepharose beads (GST pull-down; upper panel). 3xFLAG- and GST-tagged proteins were detected by immunoblotting. (**C**) HEK-293T cells were co-transfected with expression vectors encoding GST alone or fused to TioV-V, MuV-V or CHIKV-nsP4 (500 ng/well), and pCI-neo-3xFLAG expression vectors encoding for 3xFLAG-tagged human STAT1 and STAT2 (150 ng/well of each vector). At 24 h post-transfection, cells were left untreated or stimulated with recombinant IFN-β at 200 IU/ml. Total cell lysates from transfected cells were prepared at 48 h post-transfection (cell lysate; middle and lower panels), and protein complexes were assayed by pull-down using glutathione-sepharose beads (GST pull-down; upper panels). 3xFLAG- and GST-tagged proteins were detected by immunoblotting. Upper and lower panels on top of figure C correspond to short and longer exposures of the same blot, respectively. (**D**) HEK-293T cells were transfected with pCI-neo-3xFLAG expression vector (1 µg/well) either empty or encoding for 3xFLAG-tagged TioV-V, MuV-V, TioV-P or TioV-W. Total cell lysates were prepared at 48 h post-transfection and endogenous STAT1 expression levels were determined by western-blot analysis. Actin expression was determined and used as a protein extraction and loading control. (**E**) HEK-293T cells were transfected with pCI-neo-3xFLAG expression vector (1 µg/well) either empty or encoding for 3xFLAG-tagged TioV-V, MuV-V or CHIKV-nsP4. Total cell lysates were prepared at 48 h post-transfection, and 3xFLAG-tagged viral proteins were purified using anti-FLAG antibodies conjugated to sepharose beads. Co-immunopurification of endogenous STAT2 with 3xFLAG-tagged viral proteins was determined by western-blot analysis (top and middle panel, respectively). Actin expression was determined prior to the immunoprecipitation on total cell lysates and used as a protein extraction control (lower panel).

Then, we tested TioV-V binding to human STAT2 and STAT1. GST-tagged TioV-V or MuV-V were co-expressed in HEK-293T cells with expression vectors encoding for 3xFLAG-tagged STAT2 or STAT1. After 48 h, viral proteins were purified with glutathion-sepharose beads, and 3xFLAG-tagged STAT2 or STAT1 were revealed by western-blot analysis. As shown in Figure 7A, TioV-V is severely affected for its capacity to bind STAT2 when compared to MuV-V. Furthermore, neither TioV-V nor MuV-V interacted with STAT1 whereas Nipah Virus V protein (NiV-V) was able to do so as expected (Figure 7B). We also determined if TioV-V could interact with STAT2 when STAT1 and STAT2 were co-expressed. TioV-V showed no interaction with STAT1 and some very limited binding to STAT2 as reported above, whereas no interaction was detected with the GST protein alone or a control protein corresponding to nsP4 from chikungunya virus (CHIKV) (Figure 7C). In contrast, MuV-V strongly interacted with STAT2 as expected. In agreement with literature, analysis of total cell lysates showed that MuV-V expression induced degradation of 3xFLAG-tagged STAT1 in this system. Interestingly, this degradation was strictly dependent on STAT2 co-expression (compare cell lysates in Figure 7B and 7C), thus corroborating reports showing that STAT1 degradation by MuV-V is dependent on STAT2 binding. In contrast, TioV-V expression showed no effect on STAT1 or STAT2 expression levels (Figure 7C). Finally, we determined if this interaction profile was modified when stimulating IFN signaling with recombinant IFN-β. As shown in Figure 7C, we did not observe any significant changes, except that MuV-V now co-purified minimal amounts of STAT1. This could be explained by the formation of stable phospho-dependent STAT1/STAT2 dimers that co-purified with MuV-V.

We then investigated the induction of STAT1 degradation and the binding of TioV-V or MuV-V to endogenous STAT2. HEK-293T cells were transfected with expression vectors encoding for 3xFLAG-tagged TioV-V, TioV-P, TioV-W or MuV-V, and endogenous STAT1 expression was determined by western-blot analysis at 48 h post-transfection. Results showed that MuV-V efficiently induced STAT1 degradation whereas TioV-V, alike TioV-P or TioV-W, had no effect (Figure 7D). To determine TioV-V binding to endogenous STAT2, HEK-293T cells were transfected with expression vectors encoding for 3xFLAG-tagged TioV-V, MuV-V or CHIKV-nsp4, and tagged viral proteins were immunoprecipitated with anti-FLAG antibodies conjugated to sepharose beads. Endogenous STAT2 was found to co-immunoprecipitate with MuV-V but not TioV-V or control protein nsP4 (Figure 7E).

**Figure 8:**
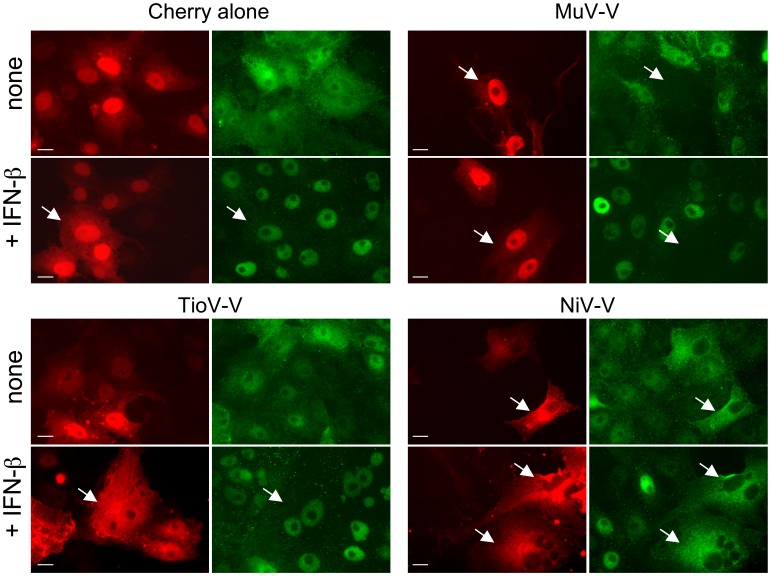
TioV-V does not inhibit STAT1 nuclear translocation induced by IFN-β. Vero cells were transfected with 100 ng of each plasmid encoding Cherry alone or fused to TioV-V, MuV-V or NiV-V. After 48 h of culture, cells were stimulated with IFN-β for 30 min, and STAT1 was labeled by immunostaining to determine its subcellular localization pattern. Green color corresponds to STAT1 whereas red corresponds to Cherry alone or Cherry-tagged viral proteins. Data show representative fields for each culture condition, and white arrows indicate cells expressing Cherry or Cherry-tagged viral proteins. Scale bar  = 10 µm.

Finally, we determined if nuclear translocation of STAT1 upon IFN-β stimulation was impaired by TioV-V expression. To address this question, we built expression vectors encoding for Cherry protein alone or fused to TioV-V, MuV-V or NiV-V. We verified that that Cherry-tagged MuV-V and NiV-V inhibited ISRE-luciferase induction by recombinant IFN-β, whereas TioV-V was unable to do so (data not shown). Constructs were transfected in Vero cells and after 48 hours of culture, cells were stimulated for 30 min with IFN-β. Vero cells were used because, in contrast to HEK-293T cells, cytosplamic and nuclear compartments are clearly distinguished, and STAT1 subcellular localization is easily visualized by immunostaining and fluorescence microscopy. As shown in Figure 8, IFN-β induced STAT1 nuclear translocation in cells expressing either Cherry alone or fused to TioV-V. This experiment was also performed in HEK-293T and A549 cells, with the same result (data not shown). In contrast and as expected, Cherry-tagged MuV-V induced STAT1 degradation as assessed by the negative immunostaining, whereas Cherry-tagged NiV-V sequestered STAT1 in the cytoplasmic compartment [35], [36]. Thus TioV-V is incapable of blocking STAT1 nuclear translocation in cells stimulated with IFN-β.

Altogether, this demonstrates that in contrast to MuV-V, TioV-V is incapable of interacting with STAT2 or STAT1, does not induce their degradation, and does not prevent STAT1 nuclear translocation upon IFN-β stimulation. These data provide molecular basis for the inability of TioV-V to block cell signaling downstream of IFN-α/β receptor in human cells.

### TioV-V Hardly Interacts with STAT2 from Giant Fruit Bat

Although this is clearly not the only parameter involved, it has been previously shown that STAT2 binding is a host restriction factor for parainfluenza virus 5 (PIV5), another *Paramyxoviridae* belonging to *Rubulavirus* genus. Indeed, the V protein of PIV5 efficiently binds STAT2 from human and other primates, but fails to interact with mouse STAT2 so that STAT1 degradation is not induced and IFN signaling is not blocked [37], [38], [39], [40]. A recombinant PIV5 expressing a mutant V protein (N100D), which binds mouse STAT2 and blocks IFN signaling but is still unable to target STAT1 for degradation, better replicates in mouse cells [37], [38], [39]. However, this does not seem sufficient to provide a selective advantage *in vivo* [40]. In contrast, it has been shown that PIV5 replicates much better in mice transgenic for human STAT2, a model where both STAT2 binding and induction of STAT1 degradation by the V protein of PIV5 is restored [39]. Altogether, this illustrates the role of STAT2 binding as host restriction factor.

We thus hypothesized that TioV-V, which failed to bind human STAT2 (hereafter “hSTAT2”), could nevertheless interact with STAT2 from giant fruit bats of *Pteropus* genus. This question was particularly interesting because STAT2 sequence from giant fruit bats has never been established. TioV was originally isolated from *Pteropus hypomelanus*, and virus-specific antibodies were also found in *P. conspicillatus, P. rufus* as well as *Rousettus madagascariensis* [5], [41], [42]. Genomic sequence for *P. hypomelanus* has not been established yet, but we had the opportunity to access blood samples from closely related *P. rodricensis* since a large colony of this endangered species is maintained in a french zoo at La Palmyre (Charente-Maritime, France). From total RNA extracts, we were able to amplify, clone and establish the sequence of STAT2 from *P. rodricensis* (_Pr_STAT2). A draft of *Pteropus vampyrus* genome has also been established by the Human Genome Sequencing Center, the Baylor College of Medicine and the Broad Institute, and is available on Ensembl database. A prediction for STAT2 sequence of this species is provided (_Pv_STAT2; ENSPVAP00000007175), but required some improvements because sequencing coverage, accuracy, and gene structure annotation were imperfect. We used our data from *P. rodricensis* together with human and other mammalian sequences to correct and improve the current prediction for _Pv_STAT2 (see Material and Methods).

**Figure 9:**
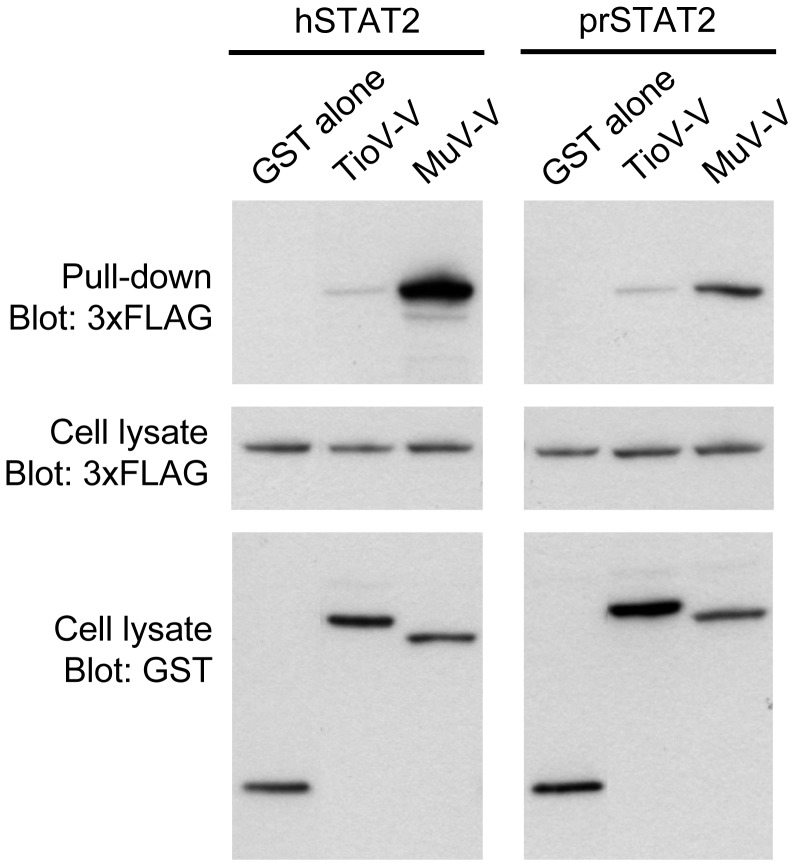
TioV-V fails to interact with _Pr_STAT2. HEK-293T cells were co-transfected with expression vectors encoding GST alone or fused to TioV-V or MuV-V (500 ng/well), and pCI-neo-3xFLAG expression vectors (300 ng/well) encoding for 3xFLAG-tagged STAT2 from human (hSTAT2) or *Pteropus rodricensis* (_Pr_STAT2). Total cell lysates from transfected cells were prepared 48 h post-transfection (cell lysate; middle and lower panels), and protein complexes were assayed by pull-down using glutathione-sepharose beads (GST pull-down; upper panel). 3xFLAG- and GST-tagged proteins were detected by immunoblotting.

As shown in Figure S1, _Pr_STAT2 and _Pv_STAT2 were almost identical, suggesting that STAT2 sequence is highly conserved among giant fruit bats of *Pteropus* genus. We thus tested the capacity of TioV-V to interact with _Pr_STAT2. GST-tagged TioV-V or MuV-V were co-expressed with 3xFLAG-tagged hSTAT2 or prSTAT2 in HEK-293T cells, and then tested for their ability to interact by co-affinity purification (Figure 9). TioV-V interaction with hSTAT2 was extremely weak as previously shown in Figure 7A. Similar results were obtained when TioV-V interaction with _Pr_STAT2 was tested. In contrast, MuV-V showed a strong capacity to bind hSTAT2, and a significant although weaker capacity to bind _Pr_STAT2. This demonstrates that unlike MuV-V, TioV-V is unable to strongly interact with STAT2 from either human or giant fruit bats.

## Discussion

We started this work showing that TioV infection activates IFN signaling in human HEK-293 cells. This suggested that TioV is incapable of blocking IFN signaling, but also fails to control IFN production. This appears surprising since TioV-V protein expression was previously shown to control IFN induction by MDA5 and RIG-I through interactions with MDA5 and LGP2, respectively [10], [13]. Although this will need to be confirmed in TioV-infected cells, our data also validated TioV-V binding to MDA5 and LGP2, and its capacity to block IFN-β promoter induction upon MDA5 overexpression. Nevertheless, IFN secretion by TioV-infected cells could be explained by several non-exclusive mechanisms. First, inhibition of RIG-I signaling by V proteins form paramyxoviruses is dependent on LGP2 recruitment, and since levels of this co-factor are rate limiting in HEK-293 cells, TioV-infection could induce IFN expression because LGP2 is not sufficiently expressed in these cells [13]. It is also possible that TioV replication produces such high amounts of a specific viral PAMP, like defective-interfering RNA genomes, that inhibition by TioV-V is ineffective. In addition, TioV could trigger IFN expression through viral sensors that are distinct from MDA5 and RIG-I. Finally, and since many cellular components involved in viral sensing are IFN-inducible, it is possible that small amounts of IFN produced at initial phases of the infection can translate into a robust expression of IFN-inducible genes at later time-points if IFN signaling is not blocked. This is supported by observations performed on PIV5 mutants (CPI- or rSV5-P/V-CPI-) expressing a V protein defective for STAT1 degradation and inhibition of IFN-α/β signaling, but competent for MDA5/LGP2 binding [43], [44]. These mutants were found to induce some more (CPI-) or much more (rSV5-P/V-CPI-) IFN-α/β than the wild-type virus [45], [46]. Since we now established that TioV-V is unable to block IFN signaling as well, this clearly parallels our observations on TioV.

Here, we demonstrate that in contrast to the V protein of MuV, TioV-V hardly interacts with human STAT2, does not induce STAT1 degradation and is unable to block signal transduction downstream of IFN-α/β and IFN-λ receptors. However, TioV-V remains functional regarding other known activities of rubulavirus V proteins, and besides interactions with MDA5 and LGP2, it was found to bind DDB1 and STAT3. This later interaction probably accounts for TioV-V capacity to block IL-6 signaling, although it was not as efficient as MuV-V to block this signaling pathway. Conformational constraints and/or a lack of adaptation to human STAT3 could explain the lower capacity of TioV-V to induce a functional complex targeting STAT3. In future, further investigations should establish that IL-6 signaling is actually impaired in TioV-infected cells.

The observation that TioV-V is unable to block IFN signaling in human cells is of interest to address ecological and epidemiological questions. Indeed, several viruses from *Paramyxoviridae* family originating from giant fruit bats have recently emerged in human populations in Southeast Asia and Australia, which include two members of *Henipavirus* genus, Hendra and Nipah viruses, and one rubulavirus closely related to Tioman virus called Menangle virus. The seroprevalence of henipaviruses (Nipah or Hendra virus) and rubulaviruses (Tioman or Menangle) in giant fruit bats can be very high (>50%) as assessed by a recent survey in Papua New Guinea [41]. Although Hendra virus has been responsible for limited outbreaks in Australian horse farms and few fatal human cases [47], Nipah virus has killed hundreds of people since 1999, while spreading from Malaysia to Singapore and Bengladesh [4]. Menangle virus also recently emerged from bats, causing disease outbreaks in Australian piggeries in 1997 with epidemiological evidence suggesting that it was responsible for severe flu-like syndromes in two piggery workers [48]. This illustrates the threat that bat *Paramyxoviridae* represent for human populations.

The V proteins of Nipah and Hendra viruses have been shown to block IFN-α/β signaling in several species including human [35], [36]. Because the V protein is essential for rubulaviruses to inhibit IFN-α/β signaling [15], [16], [17], [18], [19], [20], [21], [22], [23], [24], it is surprising that TioV-V is unable to block this pathway in human cells. Interestingly, the interaction with DDB1 suggests that at some point TioV-V was capable of targeting STAT1 proteins for proteasomal degradation but has lost this capacity during evolution. Alternatively, this interaction is maintained because TioV-V targets other cellular proteins for degradation. Nevertheless, the former hypothesis is further supported by the trace interaction detected between TioV-V and STAT2. In the future, the use of point mutants as well as MuV/TioV V chimeric proteins would be suitable to define amino acid residues in TioV-V and MuV-V that determine STAT2 binding and inhibition of IFN-α/β signaling cells. This would greatly help to establish that Tioman virus has indeed lost its capacity to efficiently bind STAT2 during evolution because of only few mutations. We also tested P and W proteins of TioV for the inhibition of IFN-α/β signaling, but none of these proteins exhibited such an activity when expressed in human cells. Although TioV could express yet unidentified viral factors to interfere with IFN-α/β signaling, *in vitro* infection experiments described in this report rather suggest some constitutive defect in the capacity of this virus to block IFN-α/β signaling in human cells.

Whether TioV is able to block IFN-α/β signaling in bats also remains a pending question. In this report, we established the sequence of STAT2 from *Pteropus rodricensis*, and then demonstrated that TioV-V is a poor binder of prSTAT2. Since this experiment was performed in human cells, it is possible that TioV-V and prSTAT2 failed to interact because bat-specific factors were missing in this microenvironment. It is also possible that TioV-V directly interacts with STAT1 from bats alike the V protein of hPIV2 [23], [25], [26], [27]. In order to determine if TioV-V can block IFN-α/β signaling in bats, experiments must be performed in cells isolated from giant fruit bats of *Pteropus* genus. Unfortunately, no commercial cell line is currently available and only one lab in Australia has developed this kind of tool [49]. Using this system, Virtue and collaborators have shown that interferon production and signaling pathways are antagonized during henipavirus infection of fruit bat cell lines [50].

Altogether, our observations question the capacity of TioV to appropriately control IFN-α/β signaling in human and bat cells. Inhibition of IFN-α/β signaling may not be mandatory for TioV to be maintained in its natural host population and to infect human, regardless the induction of a specific pathology. Indeed, it has been reported that the V protein of human parainfluenza virus type 4 (hPIV4), a member of *Rubulavirus* genus infecting human, is unable to inhibit IFN-α/β signaling in host cells [51]. This natural defect could account for the fact that hPIV4 infection is less frequent and pathogenesis is less severe compared to other human paramyxoviruses [52]. Similarly, a human parainfluenza virus type 2 (hPIV2) with V protein mutations that prevented the virus from inhibiting IFN-mediated signaling maintained its capacity to replicate in the respiratory tract of non-human primates [53]. In both cases, the capacity to block IFN-α/β induction through interactions with MDA5 and LGP2 probably compensates to some point for the lack of inhibition downstream of IFN-α/β receptor and the same could be true for TioV *in vivo* [54]. However, the capacity of *Paramyxoviridae* to target STAT proteins has also been associated to their replication level in a specific host as aforementioned for PIV5 infection in mice [37], [38], [39], but also to the development of pathology. For example, a recombinant measles virus that is unable to fully antagonize IFN signaling cannot control inflammation and is attenuated in rhesus monkeys [55]. These data demonstrate that in some cases, the ability of *Paramyxoviridae* to block this antiviral pathway directly influences their infectivity, virulence and associated pathogenesis. Since our findings suggest that TioV is defective for the inhibition of IFN-α/β signaling at least in human cells, future studies should determine consequences in terms of infectivity, virulence and pathogenesis.

## Materials and Methods

### Cell Cultures and Tioman Virus Infection

All cells were maintained in Dulbecco's modified Eagle's medium (DMEM; Gibco-Invitrogen) containing 10% fetal calf serum (FCS), penicillin, and streptomycin at 37°C and 5% CO2. Tioman virus derived from bat urine (TioV; kindly provided by Prof. S.K. Lam, University of Malaya, Malaysia) was produced on VERO-E6 cells, and titrated by plaque assay using the same cell line. Measles virus stock (MeV; strain Schwarz) was produced on VERO cells (ATCC), and titrated by TCID50 on HEK-293T cells (ATCC).

STING-37 cell line that corresponds to HEK-293 cells was stably transfected with an ISRE-luciferase reported gene, which will be described in details elsewhere (Lucas-Hourani M. & al., manuscript in preparation). Briefly, the ISRE-luciferase reporter gene was amplified by PCR from pISRE-luciferase reporter plasmid (Stratagene, Ref 219089), and inserted in a plasmid carrying a G418-resistance selection marker. This new plasmid was transfected in HEK-293 cells (ATCC) and two days later, culture medium was supplemented with G418 at 500 µg/ml. Transfected cells were amplified and subsequently cloned by serial limit dilution. A total of 44 clones were screened for luciferase expression, and STING-37 clone was selected for its optimal signal to background ratio when stimulated or not with recombinant IFN-β.

The stock of TioV used in this study was obtained in the BSL-4 “Jean Mérieux” (Lyon, France) in 2001, since the pathogenicity of this new virus was not known. Since then, the official classification has still not being made for this virus in France. According to current rules in the BSL-4 “Jean Mérieux”, this stock of TioV cannot be taken out of the BSL-4 and consequently all experiments with live TioV were performed in these stringent conditions.

STING-37 cells were trypsinized and infected in suspension with a MOI of 1 or 0.1 at 37°C in DMEM either with TioV or MeV. After 1 h of incubation, cells were centrifuged and the supernatant was removed. Cells were resuspended in DMEM +5% FCS and plated in 96-well plates. 48 h later, luciferase activity was determined by addition of 50 µl/well of Bright-GLO reagent (Promega) and measured during 0.1 s with a luminometer (Tecan).

### Plasmid DNA Constructs

TioV-V (NP_665866), TioV-P (NP_665865), TioV-W (NP_665867), TioV-VCT (AA 150-228 of TioV-V), MuV-V (ABG48763, Mumps virus isolate “Sophie”, GenBank accession number: DQ660370) and NiV-V (NP_112023.1) coding sequences were amplified by RT-PCR (Titan One tube; Roche) from RNA samples kindly provided by Dr. TF Wild, and cloned into pDONR207 (Invitrogen) using an *in vitro* recombination-based cloning system (Gateway system; Invitrogen) as previously described [30]. Chikungunya virus (CHIKV) nsP4 construct was previously described [30]. Corresponding constructs were stored in ViralORFeome database under reference IDs 493, 768, 842, 819, 217, 840 and 716 for MuV-V, TioV-V, TioV-P, TioV-W, TioV-VCT, NiV-V and CHIKV-nsP4, respectively. Compared to reference sequences in GenBank, TioV-P and TioV-W sequences from TioV strain used in this study showed amino acid mutations at positions N182S/A202V and M182V/R202W, respectively. Plasmids containing human STAT1 and STAT2 were previously described [56] whereas MDA5, LGP2, STAT3, and DDB1 coding sequences were amplified by PCR from a human spleen cDNA library (Invitrogen) before cloning into pDONR207 (Invitrogen). Subsequently, viral or cellular ORFs were transferred by an *in vitro* recombination from pDONR207 in different Gateway-compatible destination vectors (see below) following manufacturer's recommendation (LR cloning reaction, Invitrogen). To perform yeast two-hybrid experiments, TioV-V viral ORF was transferred into pDEST32 (Invitrogen) to be expressed in fusion downstream of the DNA binding domain of Gal4 (Gal4-DB). In mammalian cells, GST-tag and 3xFLAG-tag fusions were achieved using pDEST27 (Invitrogen) or pCI-neo-3xFLAG vector, respectively [57]. Expression of untagged proteins was achieved using a modified pCI-neo vector (Promega) compatible with the Gateway system (kindly provided by Dr. Yves Jacob).

DNA fragment encoding for _Pr_STAT2 was generated from total RNA purified from *Pteropus rodricensis* blood. Samples were obtained as part of a routine medical check up performed on animals of a bat colony maintained in La Palmyre zoo. Blood samples were collected by the veterinarian in charge following appropriate guidelines to minimize animal stress and suffering. Bats were anesthetized prior to be handled using a mask with isoflurane at 5% for induction and at 3% for maintenance. Blood was taken from the median vein with a 23G needle. Total RNA were purified using the RNeasy Protect Animal Blood Kit (Qiagen), transcribed into cDNA by RT-PCR, and then cloned in pDONR207 vector. Once established and deposited in GenBank (ID: JQ846265), _Pr_STAT2 sequence was aligned and compared to _Pv_STAT2 (Figure S1) sequence prediction available on Ensembl database (ENSPVAP00000007175). Two peptides of _Pr_STAT2 were absent from _Pv_STAT2: LIWDFSYL (AA 398-405) and ELKLEPILGP (AA 778-787). Furthermore, PL and NL residues of _Pr_STAT2 (AA 754-755 and 774-775) were respectively replaced by a single T and DQ in _Pv_STAT2. All these discrepancies were cleared when Ensembl splicing model for _Pv_STAT2 sequence was manually curated for intron-exon junctions. Besides, four amino acid residues from _Pr_STAT2 were either absent (P and G at positions 13 and 850, respectively) or different (P, E, P and P at positions 28, 837, 843 and 854, respectively) in _Pv_STAT2 sequence. However, all six residues are mostly if not totally conserved in human and other mammals thus suggesting that differences between _Pr_STAT2 and _Pv_STAT2 correspond to sequencing errors in _Pv_STAT2 due to the low sequencing coverage of this genome. Finally, only one amino acid substitution (A183V) likely corresponds to a genuine difference between _Pr_STAT2 and _Pv_STAT2 sequences.

### Luciferase Reporter Gene Assay

HEK-293T cells were plated in 24-well plates (2×10^5^ cells per well). One day later, cells were transfected with either IFN-β-pGL3 (0.3 µg/well; [58]) or pISRE-Luc (0.3 µg/well; Stratagene) or pSTAT3-Luc plasmids (0.3 µg/well; SABiosciences) together with pRL-CMV reference plasmid (0.03 µg/well; Promega). Cells were simultaneously co-transfected with 0.3 µg/well of pCI-neo-3xFLAG expression vectors encoding 3xFLAG alone or fused to indicated proteins. Alternatively, cells were co-transfected with 0.3 µg/well of pCI-neo expression vectors encoding indicated proteins without any tag. Transfections were performed with either Lipofectamine 2000 (Invitrogen) or JetPrime PEI (Polyplus transfection). 24 h after transfection, cells were stimulated with IFN-β (Biosource) at 200 IU/ml, 10 ng/ml of recombinant IL-6 (Miltenyi Biotech) or IFN-λ1 at 50 ng/ml (Acris). 24 h later, cells were lysed, and both firefly and Renilla luciferase activities in the lysates were determined using the Dual-luciferase Reporter Assay System (Promega). Reporter activity was calculated as the ratio of firefly luciferase activity to reference Renilla luciferase activity.

### Quantitative RT-PCR Analysis

HEK-293T cells were plated in 24-well plates (2×10^5^ cells per well). One day later, cells were transfected with 0.5 µg/well of pCI-neo-3xFLAG expression vectors encoding 3xFLAG alone or fused to indicated proteins. Transfections were performed with JetPrime PEI (Polyplus transfection). 24 h after transfection, cells were stimulated with IFN-β (Biosource) at 200 IU/ml. 24 h later, cells were recovered in PBS and total RNA isolated with the RNeasy Mini Kit (Qiagen) according to manufacturer’s protocol. Following elution, RNA yields were evaluated using a Nanodrop spectrophotometer (Nanodrop technologies).

A two-step qRT-PCR (Taqman technology, Applied Biosystems) was performed to measure transcription levels for 11 genes of interest (primer references are indicated between brackets): IFI27 (Hs00271467_m1), IFI35 (Hs00413458_m1), IFI44 (Hs00197427_m1), IFI6 (Hs00242571_m1), IFIH1 (Hs01070332_m1), IFIT1 (Hs01911452_s1), IFIT3 (Hs01922752_s1), IFITM1 (Hs00705137_s1), ISG15 (Hs01921425_s1), MX1 (Hs00895608_m1) and OAS1 (Hs00973637_m1). Expression levels of four housekeeping genes, including 18S (Hs99999901_s1), GAPDH (Hs99999905_m1), HPRT1 (Hs99999909_m1) and GUSB (Hs99999908_m1), were also determined and used as an internal reference controls. Starting from 1 µg of total RNA, cDNA synthesis was achieved in 20 µL using the SuperScript VILO cDNA Synthesis Kit following manufacturer’s recommendations (Life Technologies). Quantitative PCR reactions were performed on 0.6 µL of cDNA synthesis reaction mix using the TaqMan Fast Advanced Master Mix (Applied Biosystems) on a StepOnePlus™ Real-Time PCR machine (Applied Biosystems). Results were normalized using expression levels of the four housekeeping genes.

### Yeast Two-hybrid Screening Procedure

Our yeast two-hybrid protocol has been described in details elsewhere [59]. Briefly, pDEST32 plasmid encoding Gal4-DB fused to TioV-V was transformed in AH109 yeast strain (Clontech), and used to screen by mating a human spleen cDNA library cloned in the Gal4-AD pPC86 vector (Invitrogen) and previously established in Y187 yeast strain (Clontech). Yeast cells were plated on a selective medium lacking histidine and supplemented with 80 mM 3-amino-triazole (3-AT; Sigma-Aldrich) to select for interaction-dependent transactivation of HIS3 reporter gene. AD-cDNAs from [His+] colonies were amplified by PCR and sequenced to identify the host proteins interacting with TioV-V.

### Co-affinity Purification Experiments and Western Blot Analysis

To perform co-affinity purification experiments, cloned ORFs were transferred from pDONR207 to pDEST27 expression vector (Invitrogen) to achieve GST fusion, and to pCI-neo-3xFLAG vector for 3xFLAG fusion. Then, tagged proteins were expressed by transient transfection in HEK-293T cells. Briefly, 5×10^5^ HEK-293T cells were dispensed in each well of a 6-well plate, and transfected 24 h later with 500 ng of each pDEST27 plasmid encoding viral ORFs and 300 ng of pCI-neo-3xFLAG vector containing 3xFLAG-tagged indicated proteins. Two days after transfection, HEK-293T cells were washed in PBS, then resuspended in lysis buffer (0.5% Nonidet P-40, 20 mM Tris–HCl at pH 8, 120 mM NaCl and 1 mM EDTA) supplemented with Complete Protease Inhibitor Cocktail (Roche). Cell lysates were incubated on ice for 20 min, then clarified by centrifugation at 14,000×g for 10 min. For pull-down analysis, 400 µg of protein extracts were incubated for 1 h at 4°C with 25 µl of glutathione-sepharose beads (Amersham Biosciences) to purify GST-tagged proteins. Beads were then washed 3 times in ice-cold lysis buffer and proteins were recovered by boiling in denaturing loading buffer (Invitrogen). Purified complexes and protein extracts were resolved by SDS-polyacrylamide gel electrophoresis (SDS-PAGE) on 4–12% NuPAGE Bis–Tris gels with MOPS running buffer (Invitrogen), and transferred to a nitrocellulose membrane. Proteins were detected using standard immunoblotting techniques. 3xFLAG- and GST-tagged proteins were detected with a mouse monoclonal HRP-conjugated anti-3xFLAG antibody (M2; Sigma-Aldrich) and a rabbit polyclonal anti-GST antibody (Sigma-Aldrich), respectively.

### Detection of Endogenous STAT Proteins and co-immunoprecipitation Experiment

HEK-293T cells were plated in 6-well plates at 5×10^5^ cells per well, and transfected 24 h later with 1 µg of pCI-neo-3xFLAG expression vector encoding viral ORFs. Two days after transfection, HEK-293T cells were washed in PBS, then resuspended in lysis buffer (0.5% Nonidet P-40, 20 mM Tris–HCl at pH 8, 120 mM NaCl and 1 mM EDTA) supplemented with Complete Protease Inhibitor Cocktail (Roche). Cell lysates were incubated on ice for 20 min, then clarified by centrifugation at 14,000×g for 10 min, and analyzed by Western-blot for STAT1 expression using an anti-STAT1 monoclonal antibody at a 1∶1000 dilution (Clone 1, BD Biosciences). To control for protein extraction, β-actin expression was determined in parallel on the same samples using an anti-actin monoclonal antibody (Clone AC-15, Sigma-Aldrich).

For co-immunoprecipiation experiments, 400 µg of protein extracts were incubated for 1 h at 4°C with 20 µl of sepharose beads conjugated to M2 anti-3xFLAG monoclonal antibody (EZview Red anti-FLAG M2 Affinity Gel, Sigma-Aldrich) to purify 3xFLAG-tagged viral proteins. Beads were then washed 3 times in ice-cold lysis buffer and proteins were recovered by boiling in denaturing loading buffer (Invitrogen). Purified complexes and protein extracts were resolved by SDS-polyacrylamide gel electrophoresis (SDS-PAGE) on 4–12% NuPAGE Bis–Tris gels with MOPS running buffer (Invitrogen), and transferred to a nitrocellulose membrane. Endogenous STAT2 protein expression was determined by immunoblotting using an anti-STAT2 monoclonal antibody at a 1∶250 dilution (Clone 22, BD Biosciences). To control for protein extraction, β-actin expression was determined in parallel on total cell lysates using an anti-actin monoclonal antibody (Clone AC-15, Sigma-Aldrich).

### STAT1 Immunostaining and Subcellular Localization

To perform subcellular localization experiments, cloned ORFs were transferred from pDONR207 to pmCherry-C1 expression vector (Clontech) made compatible with Gateway system to achieve Cherry fusion. Vero cells were plated in poly-L-lysine μ-slide 8 well (Ibidi) at 10^4^ cells/well. After 24 h, cells were transfected with 100 ng of each plasmid using Lipofectamine 2000 following manufacturer’s recommendations (Invitrogen). After 48 h, cells were incubated with 500 IU/ml of IFN-β for 30 min at 37°C. Cells were fixed with PFA 3.2% for 20 min, washed and permeabilized with PBS+0.05% Triton for 5 min at room temperature. Cells were incubated overnight at 4°C with PBS+5% goat serum. Immunostaining was performed for 1 h with mouse anti-STAT1 monoclonal antibody 9H2 (Cell Signaling) diluted in PBS +5% goat serum at 1∶100. Cells were washed and stained with anti-mouse Alexa Fluor 488-conjugated secondary antibody (Invitrogen). Finally, cells were stained DAPI for 5 min, washed and aqueous mounting medium was added (Fluoromount, Sigma-Aldrich). Slides were analyzed with a fluorescence microscope (Leica DM/IRB) using oil immersion and a 40× objective.

## Supporting Information

Figure S1 **Analysis of STAT2 sequences from giant fruit bats in comparison with other mammals.** (**A**) Amino acid sequences from _Pr_STAT2 (established in this study) and _Pv_STAT2 (both before and after editing; ★indicates specific positions discussed in the Material and Methods section) have been aligned to human, panda, ferret, dog, horse, pig and cow orthologous sequences. Alignment was performed using CLC Workbench 4.0.1. Mismatch and gaps are indicated by red gradation.
(PDF)
